# SARS-CoV-2 N protein potentiates host NPM1-snoRNA translation machinery to enhance viral replication

**DOI:** 10.1038/s41392-022-01210-9

**Published:** 2022-10-08

**Authors:** Hui Wang, Danrong Shi, Penglei Jiang, Zebin Yu, Yingli Han, Zhaoru Zhang, Peihui Wang, He Huang, Hangping Yao, Pengxu Qian

**Affiliations:** 1 Center of Stem Cell and Regenerative Medicine, and Bone Marrow Transplantation Center of the First Affiliated Hospital, Zhejiang University School of Medicine, Hangzhou, China; 2 Liangzhu Laboratory, Zhejiang University Medical Center, 1369 West Wenyi Road, Hangzhou, China; 3 Institute of Hematology, Zhejiang University & Zhejiang Engineering Laboratory for Stem Cell and Immunotherapy, Hangzhou, China; 4 School of Medicine, Zhejiang University, Hangzhou, China; 5 State Key Laboratory for Diagnosis and Treatment of Infectious Diseases, National Clinical Research Center for Infectious Diseases, Collaborative Innovation Center for Diagnosis and Treatment of Infectious Diseases, The First Affiliated Hospital, School of Medicine, Zhejiang University, Hangzhou, China; 6 Advanced Medical Research Institute, Cheeloo College of Medicine, Shandong University, Jinan, China

**Keywords:** Non-coding RNAs, Infectious diseases, Epigenetics

**Dear Editor**,

COVID-19 (Coronavirus Disease 2019) is causing an unprecedented public health crisis. Protein translation is crucial for virus lifecycle. Nucleocapsid (N) protein is among the most abundant SARS-CoV-2 proteins and highly conserved across coronavirus genus.^1^ However, its function in subverting host translation machinery is still elusive.

To explore crucial signaling pathways for SARS-CoV-2 lifecycle, we initially analyzed expression profiles of ACE2, CTSL and TMPRSS2 in susceptible organs (Supplementary Fig. S1a–f). Meanwhile, GSEA and GO term analysis revealed that translation related pathways were significantly enriched among SARS-CoV-2 infection-related genes in these organs (Supplementary Figs. S1g–1I, S2a). These results suggested that host cell translation machinery was crucial during SARS-CoV-2 invasion. Besides, host proteins interacting with N protein were enriched in “translation” and “ribosome” related pathways^2^ (Fig. 1a). Therefore, we next focused on N protein and its impact on host translation machinery.

**Fig. 1:**
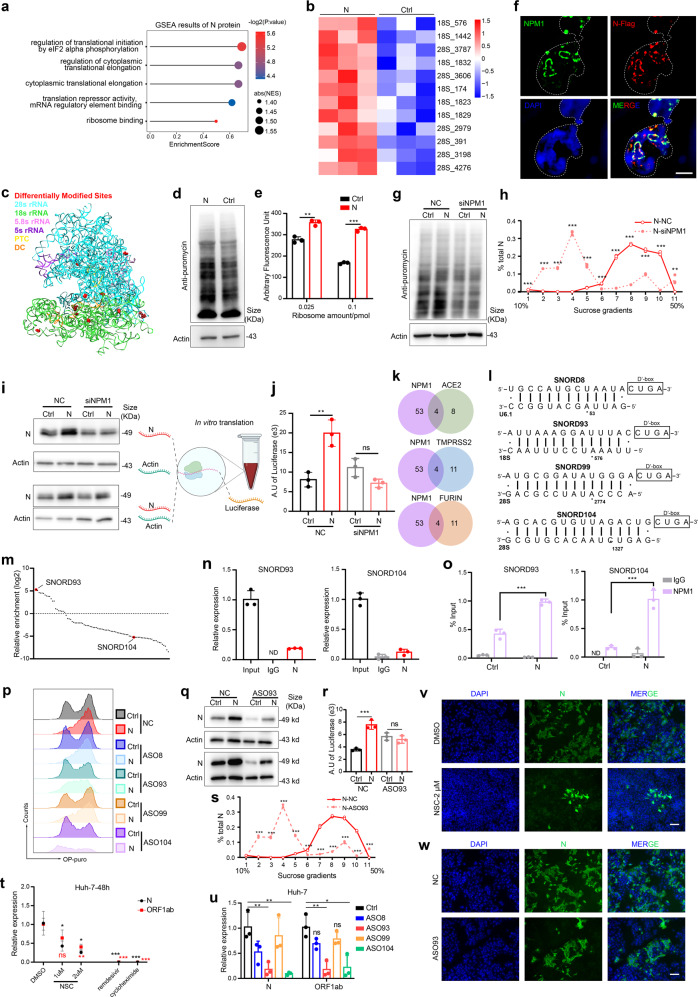
N protein potentiates host NPM1-snoRNA translation machinery to enhance viral replication. **a** GSEA analysis was performed on proteins that were interacted with N protein. **b** Heatmap of differentially methylated 2′-O-Me sites on rRNAs with or without N protein expression. N protein was ectopically expressed in 293T cells followed by RiboMethSeq. Only sites with significantly different modification levels are shown (FDR < 0.05, *P* value < 0.05). **c** 3D model showing the distribution of DMS on rRNAs. DMS (shown in red and sphere mode) were denoted on the 3D structure of human rRNAs (4UG0.pdb) using UCSF Chimera software. The RNA backbone is presented in ribbon mode, and rRNAs and functional regions (PTC peptidyl-transferase center, DC decoding center) are color-coded. **d**, **e** Effect of SARS-CoV-2 N protein on host translation activity. 293T cells were transfected as in (**b**) and overall translation activity was measured using the SUnSET assay (**d**) and in vitro translation assay (**e**). **f** Validation of interactions between N and host NPM1. 293T cells were transfected with plasmid expressing N or empty vector, and 48 h later immunofluorescence staining was performed using specific antibodies recognizing Flag (N, red) and NPM1 (green). DNA was stained with DAPI (blue). Nuclear regions were outlined by dashed lines. Scale bar: 5 μm. **g** Knockdown of NPM1 attenuated translation enhancement by ectopic N expression. 293T cells were transfected with N-expressing plasmids, along with NPM1 siRNA or controls, and overall translation activity was measured using SUnSET assay. **h** Host cell translation activity was measured by polysome profiling and the distribution of N mRNA across these sucrose gradient fractions was examined by RT-qPCR and plotted under different treatment conditions (NC, siNPM1). The y axis reflects the relative amount of N mRNA in each fraction, while the x axis shows each sucrose fraction ranging between 10 and 50%. **i**, **j** Ribosome translation activity was quantified using the in vitro translation system after NPM1 knockdown. Schematic representation of experimental design is shown in (**i**) on the right. 293T cells were treated as in (**g**), and ribosomes were isolated. An in vitro translation assay was performed to quantify translation activity. Different in vitro transcribed mRNAs were added to evaluate ribosome translation efficiency for either N/actin mRNAs (**i**) or exogenous luciferase mRNA (**j**). **k** Venn diagrams show overlap between NPM1-binding snoRNAs and those with strong correlation with SARS-CoV-2 infection-related genes. **l** SnoRNA targets were predicted using snoRNA Atlas,^6^ and the sequence alignments are shown. The top of each alignment shows snoRNA sequences with the D′-box labeled, and the bottom shows their target sequences with specific 2′-O-Me sites labeled. **m** N protein-binding snoRNAs were analyzed using published data.^5^ Results were shown as relative enrichment of snoRNAs co-immunoprecipitated with N protein compared with input. NPM1-binding snoRNAs (SNORD93, SNORD104) were indicated with red circles. **n**, **o** 293T cells were transfected with N-expressing plasmids or according control vectors, RNA immunoprecipitation was performed using antibodies targeting N (**n**), NPM1 or IgG (**o**). RNA binding was quantified by RT-qPCR and shown as relative enrichment to input (10% loading). **p**–**r** 293T cells were transfected with N-expressing plasmids and snoRNA antisense oligos (ASOs), and cell translation activity was measured using OPP-Puro assay (**p**) or in vitro translation assay, using in vitro transcribed N, actin (**q**) or luciferase mRNAs (**r**). **s** 293T cells were treated as in (**p**), and polysome profiling was performed. The distribution of N mRNA across sucrose gradient fractions was assessed by RT-qPCR and plotted under different treatment conditions. **t** Huh-7 cells were infected with SARS-CoV-2 at an MOI of 0.01 and treated with NPM1 inhibitor (NSC), remdesivir, or cycloheximide, and SARS-CoV-2 genomic RNA in the culture medium was quantified by RT-qPCR at 48 hpi. **u** Huh-7 cells were infected with SARS-CoV-2 at an MOI of 0.01 and transfected with snoRNA ASOs, and SARS-CoV-2 genomic RNA in the culture medium was quantified by RT-qPCR at 48 hpi. **v**, **w** Huh-7 cells were infected and treated/transfected as in (**t**, **u**), and virus replication in host cells was observed by IF. A specific antibody recognizing SARS-CoV-2 N protein (green) was used and DNA was stained with DAPI (blue). Scale bar, 20 μm. *N* = 3 independent repeats. Results are presented as mean ± SD. Statistical significance of difference was calculated using un-paired student *t* test, and error bars indicate standard deviation (*P < 0.05; **P < 0.01; ***P < 0.001; ns non-significant)

Post-transcriptional modifications of rRNAs are involved in ribosome biogenesis and fine-tuning of translation, with the 2′-O-Methylation being the most abundant (Supplementary Fig. S2b). We first examined how N protein affects 2′-O-Me modification on host rRNAs by RiboMethSeq in HEK293T cells. We detected 12 sites on rRNA as differentially modified sites (DMS), all of which presented elevated modification upon N protein expression (Fig. 1b, c). Ectopic N protein enhanced global protein synthesis in 293T cells (Fig. 1d). and similarly, polysome fractions significantly increased, indicating mRNAs were translated at higher efficiency (Supplementary Fig. S2c). Using a hybrid in vitro translation (IVT) system (Supplementary Fig. S2d), we found that ribosomes from 293T cells with ectopic N protein showed enhanced translation efficiency (Fig. 1e). These results demonstrate that ectopic expression of N protein promoted host translation, with direct impact on rRNA modification.

We re-analyzed the interactome data between SARS-CoV-2 proteins and host proteins^2^ and discovered extensive interactions between SARS-CoV-2 proteins and host rRNA modification related complexes (Supplementary Fig. S3a). We then experimentally verified these interactions (Supplementary Fig. S3b), and found that NPM1 interacted with multiple SARS-CoV-2 proteins, which was verified by Co-IP and immunofluorescence staining (Fig. 1f and Supplementary Fig. S3b–d). We also performed ELISA assays and found that N protein bound to pre-coated NPM1 in a dose-dependent manner (Supplementary Fig. S3e, f). Biolayer interferometry assays (BLI), and Surface Plasmon Resonance (SPR) also revealed the interaction between N and NPM1 (Kd = 0.293 μM) (Supplementary Fig. S3g, h). The association between N and NPM1 was significantly attenuated by RNase A treatment, suggesting that their interaction was at least partially RNA-dependent (Supplementary Fig. S3i). We next constructed series of truncations of N protein. Only the long isoform of T3 was co-immunoprecipitated with NPM1 (Supplementary Fig. S4a) and co-localized with NPM1 (Supplementary Fig. S4b). None of the truncations were able to enhance translation efficiency comparably to full-length N protein, suggesting the importance of structural integrity to its function (Supplementary Fig. S4c). Besides, we found that other structural proteins, such as M, were neither able to enhance translation efficiency nor interacting with NPM1 (Supplementary Fig. S4d, e). These results provide strong evidence for the specific interaction between N and NPM1, and the SR motif plays an essential role in the interaction.

NPM1 is a multifunctional protein which shuttles between nucleus and cytoplasm. It is involved in many physio/pathological processes and has been reported to regulate rRNA modification and ribosome biogenesis via interacting with snoRNAs.^3^ Knockdown of NPM1 significantly suppressed translation efficiency that was boosted by N protein (Fig. 1g, Supplementary Fig. S5a, b). Besides, treatment with NPM1 inhibitor, NSC348884, also partially rescued the effect of N protein (Supplementary Fig. S5c–i).

We next quantified translation efficiency of specific genes using total RNAs extracted from separate sucrose gradients in the polysome profiling assay. Most N mRNA was presented in polysome fractions, indicating highly efficient translation, and knockdown of NPM1 led to a drastic shift of N mRNA distribution toward the monosome fractions (gradients 2–5), indicating that N mRNAs were much less efficiently translated after NPM1 depletion (Fig. 1h). Similar results were observed after NSC treatment (Supplementary Fig. S5j). Moreover, N protein also led to a much milder increase in the amount of host mRNA (actin) in polysome fractions, which decreased after NPM1 knockdown or NSC treatment (Supplementary Fig. S5k, l).

In IVT assays, ribosomes from 293T cells with exogenous N showed enhanced translation efficiency when translating N mRNAs, which was suppressed upon NPM1 knockdown (Fig. 1i, top row) or NSC treatment (Supplementary Fig. S5m, top row). However, translation of host actin mRNAs was not affected by N protein (Fig. 1i and Supplementary S5m, 2nd row). When both N and actin mRNAs were presented in one IVT reaction, partially mimicking competition between viral and host mRNAs, ribosomes still showed higher translation efficiency for N mRNAs, but not for actin, and NPM1 inhibition suppressed this effect (Fig. 1i and Supplementary S5m, bottom rows). When using exogenous mRNA (Luciferase mRNA), similar results were observed (Fig. 1j and Supplementary S5n). These results demonstrated that ectopic N protein potentiated host translation machinery via interacting with NPM1, which might predominantly enhance translation efficiency of virus transcripts.

NPM1 has been shown to function as RNA/DNA binding protein regulating 2′-O-methylation of rRNA via direct binding of snoRNAs.^3^ We therefore hypothesized that N protein functions via NPM1-binding snoRNAs. We first re-analyzed snoRNA expression in different tissues using public data from ENCODE,^4^ and found significant overlap of expression profile among lung, liver, and intestine (16/top 20, Supplementary Fig. S6a–c), of which 4 snoRNAs were reported to bind to NPM1^3^ and their expression was highly correlated with ACE2, TMPRSS2, FURIN (Fig. 1k, l, Supplementary Fig. S6d). Exploiting published data, we also found that SNORD93 was the most highly enriched, among snoRNAs bound to N protein^5^ (Fig. 1m). This was verified by RNA immunoprecipitation (Fig. 1n and Supplementary S6e). Besides, we found that N protein significantly promoted binding of snoRNAs to NPM1 (Fig. 1o and Supplementary S6f). Knockdown 3 of the 4 snoRNAs fully restored translation to normal levels (Fig. 1p, Supplementary Fig. S6g, h). Besides, another snoRNA, SNORD61, albeit not highly correlated with ACE2, also showed similar rescuing effects (Supplementary Fig. S6i–k).

SNORD93 was predicted to target A576 on 18S rRNA for 2′-O-Me modification (Fig. 1l), which increased most after ectopic expression of N (Fig. 1b), and bound to N protein with highest abundance (Fig. 1m). These data suggested that SNORD93 was a crucial downstream factor. In IVT assay, Knockdown SNORD93 significantly rescued ribosome translation activity in IVT assay (Fig. 1q, r) and recovered polysome fraction amount and mRNA distribution across the sucrose gradients (Fig. 1s, Supplementary Fig. S6l–m). Distribution of actin mRNA was affected but to much less extent (Supplementary Fig. S6o). Besides, another SARS-CoV-2 protein nsp16, the methyl-transferase, was also affected to similar extent as N protein (Supplementary Fig. S6p). Together, these results show that SNORD93 plays a crucial role in regulating the function of N protein.

We next explored strategy for SARS-CoV-2 prevention via targeting N protein-related translation machinery components. SARS-CoV-2 infection also enhanced 2′-O-Me modification, as was detected by RTL-P assay (Supplementary Fig. S7a). In Huh-7 and Calu-3 cells, exogenous N protein enhanced translation via interaction with NPM1 (Supplementary Fig. S7b–g). In SARS-CoV-2 infected Huh-7 cells, co-localization was also observed by IF staining (Supplementary Fig. S7d). NSC dose-dependently suppressed SARS-CoV-2 proliferation, although not as effective as remdesivir and cycloheximide in Huh-7 and Calu-3 cells (Fig. 1t, and Supplementary S7h–j). Besides, depletion of SNORD93 or SNORD104 significantly reduced virus proliferation in Huh-7 other than Calu-3 (Fig. 1u–w, Supplementary Fig. S7k–m). Moreover, knockdown of NPM1 in Huh-7 cells also significantly inhibited virus proliferation (Supplementary Fig. S7n–q). These results support our hypothesis that SARS-CoV-2 proliferation could be effectively attenuated by NPM1 inhibition or snoRNA ASO, nominating promising potential therapeutic strategies for clinical use.

In summary, we found that SARS-CoV-2 N protein enhances host translation efficiency by potentiating the host NPM1-snoRNA translation machinery. By interacting with NPM1 and snoRNAs, N protein significantly promotes ribosome translation efficiency, particularly for viral mRNAs, via enhancement of snoRNA-mediated 2′-O-methylation on rRNAs (Supplementary Fig. S8). Finally, targeting NPM1 or N-related snoRNAs efficiently ameliorated SARS-CoV-2 proliferation. Our work provides preliminary data in understanding the SARS-CoV-2 viral lifecycle and offers novel potential strategies for COVID-19 prevention and therapy.

## Supplementary information

Supplementary information

## Data Availability

All data reported in this paper will be shared by the lead contact upon request. The data generated through 2nd-generation sequencing during this study are available at GEO under accession number GSE199130.
