# Supplementary material for: SARS-CoV-2 N protein potentiates host NPM1-snoRNA translation machinery to enhance viral replication
Source: Signal Transduct Target Ther. 2022 Oct 8;7:356. doi: 10.1038/s41392-022-01210-9 (PMC9547094; doi:10.1038/s41392-022-01210-9)
Supplement: Supplementary file 1 — Supplementary information [file 41392_2022_1210_MOESM1_ESM.docx]

Supplementary Materials for

SARS-CoV-2 N protein potentiates host NPM1-snoRNA translation machinery to enhance viral replication

Hui Wang^1#^, Danrong Shi^2#^, Penglei Jiang^1^, Zebin Yu^1^, Yingli Han^1^, Zhaoru Zhang^1^, Peihui Wang^3^, He Huang^1^, Hangping Yao^2^*, Pengxu Qian^1^*

Correspondence to: [yaohangping@zju.edu.cn](mailto:yaohangping@zju.edu.cn); [yaohangping@zju.edu.cn](mailto:yaohangping@zju.edu.cn)

**This PDF file includes:**

Materials and Methods

Figures. S1 to S8

Tables S1 to S2

**Materials and Methods**

**Cell culture and plasmid construction**

HEK293T, Calu-3 cell line were obtained from the American Type Culture Collection (ATCC), and Huh-7 cell line was obtained from Japanese Collection of Research Bioresources. HEK293T cells were grown in DMEM supplemented with 10% fetal bovine serum at 37°C with 5% CO_2_. Calu-3 cells were grown in alpha-MEM supplemented with 10% fetal bovine serum at 37°C with 5% CO_2_. Huh-7 cells were grown in DMEM supplemented with 10% fetal bovine serum at 37°C with 5% CO_2_. Original SARS-CoV-2 protein expression plasmids were kind gifts from Prof. Wang Pei-Hui, Shandong University. Constructs expressing N protein with specific point mutations were generated by overlap PCR.

**Virus infection**

The virus (hCoV-19/Hangzhou/ZJU-05/2020, GISAID accession ID: EPI_ISL_415711) was isolated from the sputum of a COVID-19 patient, and was identified by PCR and sequencing^1^. The structure of the authentic SARS-CoV-2 virus was observed by cryoelectron tomography^2^. The virus was titrated by TCID50.

Viral infection was conducted as previously described^1^. Briefly, cells were seeded in plates 1 day prior to viral infection to reach 80–90% confluency. The PFUs/mL of the virus strain was obtained by multiplying TCID50 with the empirical coefficient 0.7 recommended by ATCC. The MOI of infection was calculated according to the number of cells per well. The cells infected with SARS-CoV-2 grown in DMEM supplemented with 5% FBS at 35℃ with 5% CO2.

**Material source and identifiers**

Source and identifiers of reagents, antibodies, commercial kits are listed in Table S1. Sequences of primers and oligonucleotides are listed in Table S1.

**RiboMethSeq**

RiboMethSeq was performed according to previous reports^3,4^. Briefly, 1 μg total RNA was subjected to alkaline hydrolysis, which was performed in 50 mM bicarbonate buffer pH 9.2 for 12 min at 95°C. The reaction was stopped by ethanol precipitation using 3M NaOAc, pH 5.2 and Glycoblue (Invitrogen, USA) as a carrier in liquid nitrogen. The size of generated RNA fragments was assessed by electrophoresis (1% agarose gel, 80 V, 50 min) and ranged from 50-200 nt.

RNA fragments were directly 3’-end dephosphorylated using 5 U Antarctic Phosphatase (NEB, UK) for 30 min at 37°C, and then phosphorylated at the 5’-end using T4 PNK and 1 mM ATP for 1 h at 37°C. Resulting RNA fragments were then purified using Rneasy MinElute Cleanup kit (QIAGEN, Germany) according to the manufacturer instructions with minor modification. Finally, 10 μL of nuclease-free water was used to elute RNA fragments.

Construction of the RNA fragment library was performed by LC Sciences (Hangzhou, China) using TruSeq Small RNA Sample Prep Kits (Illumina, USA) following manufacturer instructions. After PCR amplification, products were purified on a 6% poly-acrylamide Tris-borate-EDTA gel. DNA fragments corresponding to 140~270 bp (the length of fragments plus adaptors) were recovered and sequenced on Illumina Hiseq2500 platform and single-end 50 bp reads were generated.

Sequenced data were trimmed with trimmomatic and keep reads which length more than 17 bp. Clean data were mapped to rRNA reference (18S rRNA, 5.8S rRNA and 28S rRNA sequences were downloaded from https://www-snorna.biotoul.fr/index.php) using bowtie2 with parameter “--end-to-end -k 1”. The sam files obtained from the alignment were converted to the bed files. 5’ and 3’ ends counting was done by bedtools (2.29.0)^5^ and combined to calculate RiboMeth score. The scores were normalized to average values for -6 and +6 nucleotides. The candidate methylated sites were screened according to the RiboMeth score of at least two samples greater than 0.8. Differential methylated sites were identified by t-test with p-value < 0.05.

**Single cell transcriptome quality control, Cell Clustering and Cell type identification**

The original authors had adequately filtered the colon data, therefore we utilized it for further research. For liver data, cells with less than 100 expressed genes, less than 1500 UMI counts and more than 50% mitochondrial genome transcript were eliminated from the liver data. Genes expressed in less than three cells were eliminated. For the kidney data, we processed it according to the code and annotation files provided by the authors.

Normalization and PCA (principal component analysis) were performed in the R package Seurat^6^ using the default settings. Different data processing methods were used on different datasets, as shown below. Liver: The first 40 principal components resulted in PCA were used to perform cell clustering and non-linear dimensionality reduction (Uniform Manifold Approximation and Projection, UMAP). Colon: With default settings, R package Harmony^7^ was used to eliminate batch effects. Then we used first 40 components resulted in Harmony to perform cell clustering and non-linear dimensionality reduction as same as liver data. We could estimate which cell types the cell clusters belong to depending on the expression level of cell markers presented in the original article matching to the scRNA-seq datasets. The annotated clusters were then shown using UMAP plots with "DimPlot" function in Seurat. Normalized gene expression levels were presented in the UMAP and point plots by R package ggplot2^8^.

***In vitro* translation**

*In Vitro* translation was performed according to previous reports with minor adjustment^9,10^. Briefly, BM was first isolated and red blood cells were lysed leaving monocytes. The cell pellet was resuspended in an iso-volume of lysis buffer R (HEPES 10 mM pH7.5, KAc 10 mM, MgAC_2_ 1 mM, DTT 1 mM). Cell suspension was homogenized using Dounce homogenizer to obtain cytoplasmic lysate, which was then centrifuged at 20,000 g for 20 min at 4°C. Supernatant was then transferred to new RNase-free Eppendorf tubes, discarding the pellet which contains most of the nucleus and mitochondria. Supernatant was then subjected to super-centrifugation at 170,000g for 2.5 h. After that, pellet was washed 2 times and resuspended in buffer R2 (HEPES 20 mM pH 7.5, NaCl 10 mM, KCl 25 mM, MgCl_2_ 1.1 mM, DTT 1 mM).

Rabbit reticulocyte lysate (Promega, USA) was depleted of endogenous ribosome by centrifugation at 170,000 g for 2.5 h at 4°C, ribosome pellet was resuspended in buffer R2 as positive controls. Supernatant was used for in vitro translation reactions supplemented with ribosomes purified from mouse BM, using luciferase activity as readout for translation efficiency.

**SUnSET assay**

Translation rate was measured using the SUnSET assay according to previous report^11^. Briefly, 0.3~1 million cells were seeded onto 12-well plate and treated with puromycin (Sangon Biotech, China) at a concentration of 10 μg/mL for 1 h at 37°C in 5% CO_2_ atmosphere. Cells were then harvested, and puromycin-incorporated proteins were detected by western blot using an antibody targeting puromycin (Millipore, USA).

**OP-puro assay**

OP-puro assay was performed using Click-iT Plus OPP Protein Synthesis Assay Kit (Thermo, USA), according to user manual. Briefly, cells were treated with 10 μM OPP for 15 min at 37°C, and then fixed with 3.7% formaldehyde in PBS for 15 min at room temperature. After washing with PBS, cells were permeabilized for 15 min with 0.5% Triton X-100 in PBS and washed with PBS. Then the OPP labelled nascent proteins were attached to Alexa Fluor-488 azide dye and analyzed by flow cytometry.

**Immunofluorescence staining**

Cells grown in monolayers under were first fixed with pre-chilled acetone at 4 °C for 15 min. Then cells were washed for three times using pre-chilled PBS, and incubate cells in 1% BSA for 1 h. After that, incubate them with according antibodies (diluted in PBS with 0.1% BSA at 1:1000) at 4 °C overnight. The cells were then washed again with pre-chilled PBS for 3 times, and incubated with according fluorescent secondary antibodies (diluted in PBS with 0.1% BSA at 1:5000) for 2 h RT avoiding light. Then the cells were washed with PBS and stained with DAPI for 10 min. After final washing with PBS, cells were imaged using confocal microscope equipped with a fluorescence apparatus (OLYMPUS IX83-FV3000).

**Immuno-precipitation**

Cells lysates were prepared using TNE buffer (Tris-HCl pH=7.4, 20 mM; NaCl 150 mM; EDTA, 2 mM; NP-40, 0.5%) on ice, and then centrifuged at 12,000 g for 15 min at 4°C. Supernatants were collected and subjected to immunoprecipitation using anti-Flag or anti-NPM1 antibodies. Nuclear protein lysates were prepared using Cytoplasmic and nuclear protein extraction kit (Beyotime, China) according manufacturer instructions, and nuclear protein lysates were subjected to immunoprecipitation using anti-NPM1 antibody.

**Polysome profiling**

Approximately 1e^7^ cells were treated with cycloheximide (Millipore, USA) in media at 100 μg/mL for 15-30 min at 37°C, and then resuspended in 300 μL MCB buffer on ice for 15 min. MCB buffer: 50 mM HEPES pH 7.5, 100 mM KCl, 2 mM MgCl_2_, 1 mM DTT, 10% glycerol, 0.1% Triton X-100, 100 μg/mL cycloheximide, 0.2 U/μL. RNase inhibitor (Thermo Fisher Scientific, USA), 1×protease inhibitor cocktail (Thermo Fisher Scientific, USA). To remove nuclei, lysate was centrifuged first at 5000 rpm for 5 min and then at 15000 rpm for 10 min, each time taking the supernatants. The lysate was then layered onto a sucrose gradient (20%-50%) and ultracentrifuged on a Beckman Coulter SW-41Ti rotor at 38,000 rpm. for 165 min at 4 °C. Sucrose gradient buffer: 10 mM Tris–HCl pH 7.5, 100 mM NaCl, 5 mM MgCl2, 1 mM DTT, 100 μg/mL cycloheximide.

The gradient was density fractionated using Gradient Profiler (BioComp, Canada) into 12× 900-μL fractions. RNA of each fraction was extracted using acid phenol–chloroform, and *in vitro* transcribed luciferase RNA (50 pg) as spike-in was added. RNA (100 ng) was then reverse transcribed using HiScript II reverse transcriptase (Vazyme, China), and qPCR was performed using Universal SYBR Green Supermix (Bio-Rad).

Ct values were normalized to that of spike-in luciferase and plotted as proportions across the 12 fractions. The t-statistic and *P* value were calculated for difference between the two genotypes for each fraction.

**RNA immunoprecipitation**

RNA immunoprecipitation (RIP) was performed using commercial kit (BersinBio, China) and all solutions and reagents were supplied in the kit. In brief, 1e^7^ cells were harvested and washed in pre-chilled PBS. Then cells were pelleted and lysed using 0.9 mL polysome lysis buffer supplemented with 9 μL protease inhibitor and 4.5 μL RNase inhibitor for 15 min; Then add 4.5 μL DNase salt stock and 10 μL DNase, and incubate lysate in 37 °C water bath for 10 min; the reaction was stopped by adding 4.5 μL EDTA, 1.8 μL EGTA and 9 μL DTT; lysate was then centrifuged at 16,000 g for 10 min at 4 °C, and transfer supernatant to new RNase-free tubes. 0.8 mL lysate was subjected to incubation with according antibodies, and the remaining 0.1 mL was used as input; after incubation at 4 °C for 16 hr, 20 μL protein A/G magnetic beads was added to each sample, and incubated at 4 °C for 1 hr; beads were than collected and washed 3 times using polysome washing buffer 1 and 2 and eluted in 200 μL polysome elution buffer supplemented with 2 μL DTT, 2 μL proteinase K at 55 °C for 1 hr; RNA was extracted using phenol-chloroform methods and analyzed by RT-qPCR.

**RTL-P assay**

2’-O-Me modification levels of rRNAs at specific sites were detected according to previous report^12^. Briefly, 200 ng total RNA was reverse-transcribed at different dNTP concentrations (1 mM or 1 μM) using Hiscritpt III Reverse Transcriptase (Thermo). qPCR was then performed to detect according cDNA products using primers targeting specific 2’-O-Me modification sites (primer sequences were listed in Table S1).

**Bio-layer interferometry assay**

Biolayer interferometry assays (BLI) were performed to examine interactions between N protein and NPM1 using Octet RED96e (Pall-Fortebio, Shanghai, China) instrument. For binding kinetics assays, lyophilized N and NPM1 (Sino Biological, China) were dissolved in SD buffer (0.1% BSA, 0.05% Tween20, 10 mM PBS, pH7.4), and streptavidin probes (Satorius, Germany) were incubated with 200 μL biotinylated N protein (10 μg/mL) for 2000s. A serial dilution of seven concentrations of NPM1 solution (200 μL) in SD buffer were used for binding kinetic assay, following an assay cycle including 60s of baseline, 200s of association and 300s of dissociation. Data was analyzed using ForteBio data analysis software (V9.0).

**ELISA assay**

Microplates were coated with 100 μL NPM1 (50ng, Sino Biological) in coating buffer (InnoReagents) overnight at 4°C, then the plates were washed 3 times with washing buffer (0.05% Tween-20 in PBS) and then were blocked with blocking reagent (InnoReagents) for 2 hours at room temperature (RT). After washing, the plates were then incubated with different concentrations of SARS-CoV-2 N protein (Sino Biological) for 2 hours at room temperature. After washing again, rabbit anti-SARS-CoV-2 N protein (Abcam) were added and washed after 2 hours’ incubation. SARS-CoV-2 N protein bound to NPM1 were detected by HRP conjugated goat anti-rabbit IgG antibody (Abcam) for 1 hour at RT and washed again. Finally, plates were incubated with HRP substrate solution (InnoReagents). The absorbance was measured at 450 nm using Microplate Reader (Bio-rad).

**Surface plasmon resonance (SPR) for NPM1-N interaction assessment**

Surface plasmon resonance (SPR) for NPM1-N interaction assessment

SPR experiment was performed by Biacore X100 (GE Healthcare). First, NPM1 diluted with 10mM sodium acetate (pH=4.0) was immobilized on the CM5 sensor chips by Amine Coupling Kit (GE Healthcare). Then, N protein was diluted with pure water in a series of concentrations and flowed over the NPM1 immobilized CM5 sensor chip with 180 s contact time and 300 s dissociation time. 10mM glycine-HCl (pH=1.5) was used for dissociation. Resonance units were used to express binding results.

**Data resources and analysis**

Public single-cell [RNA sequencing](https://www.sciencedirect.com/topics/medicine-and-dentistry/rna-sequence) datasets were downloaded from the Gene Expression Omnibus (<https://www.ncbi.nlm.nih.gov/geo/>). The colon dataset used in this study was obtained from GSE116222^13^ taken from colonic biopsies collected from healthy patients. Liver data were obtained from HCA, include 5 hepatic tissues from organ donors^14^. Kidney data were obtained from GSE131685, include primary kidney samples from human kidneys of 3 donors.

To investigate the interactions specificity of the N protein of SARS-CoV-2 with the human RNAs, we analyzed the published studies SRR12712780 and SRR1271284. The former is the RNA interactome of N protein, and the latter is the total RNA-seq outcomes of N protein. We use fastq-dump, trimmomatic, hisat2, samtools to complete the upstream process from SRR files to bam files. The resulting bam files were fed to HTSeq tool to count the number of RNA-seq reads, which was further normalized to calculate FPKM. We then divided the RNA interactome of N protein by total RNA-seq outcomes to obtain the specificity of the interaction of different RNAs on the N protein of SARS-CoV-2 and performed GSEA analysis.

To analysis snoRNA expression in different organs/tissues, we downloaded the data sets from the ENCODE portal, with identifier numbers listed in Table S2.

**Statistics**

Data are presented as mean ± SD or SEM. Raw data were analyzed using GraphPad Prism 8 (GraphPad Software). Student’s t test was used for comparisons between 2 groups, whereas one-way ANOVA followed by Tukey’s post hoc tests and two-way ANOVA analysis followed by Bonferroni’s post hoc tests were used for comparisons among multiple groups. Statistical significance was defined as P<0.05.

**References:**

1 Yao, H. *et al.* Patient-derived SARS-CoV-2 mutations impact viral replication dynamics and infectivity in vitro and with clinical implications in vivo. *Cell Discov* **6**, 76, (2020).

2 Yao, H. *et al.* Molecular Architecture of the SARS-CoV-2 Virus. *Cell* **183**, 730-738 (2020).

3 Marchand, V., Blanloeil-Oillo, F., Helm, M. & Motorin, Y. Illumina-based RiboMethSeq approach for mapping of 2'-O-Me residues in RNA. *Nucleic Acids Res* **44**, e135, (2016).

4 Birkedal, U. *et al.* Profiling of ribose methylations in RNA by high-throughput sequencing. *Angew Chem Int Ed Engl* **54**, 451-455, (2015).

5 Quinlan, A. R. & Hall, I. M. BEDTools: a flexible suite of utilities for comparing genomic features. *Bioinformatics* **26**, 841-842, (2010).

6 Stuart, T. *et al.* Comprehensive Integration of Single-Cell Data. *Cell* **177**, 1888-1902 e1821, (2019).

7 Korsunsky, I. *et al.* Fast, sensitive and accurate integration of single-cell data with Harmony. *Nat Methods* **16**, 1289-1296, (2019).

8 Wickham, H. *ggplot2: Elegant Graphics for Data Analysis*. (Springer-Verlag New York, 2016).

9 Penzo, M., Carnicelli, D., Montanaro, L. & Brigotti, M. A reconstituted cell-free assay for the evaluation of the intrinsic activity of purified human ribosomes. *Nat Protoc* **11**, 1309-1325, (2016).

10 Panthu, B., Décimo, D., Balvay, L. & Ohlmann, T. In vitro translation in a hybrid cell free lysate with exogenous cellular ribosomes. *Biochem J* **467**, 387-398, (2015).

11 Schmidt, E. K., Clavarino, G., Ceppi, M. & Pierre, P. SUnSET, a nonradioactive method to monitor protein synthesis. *Nat Methods* **6**, 275-277, (2009).

12 Zhou, F. *et al.* AML1-ETO requires enhanced C/D box snoRNA/RNP formation to induce self-renewal and leukaemia. *Nat Cell Biol* **19**, 844-855, (2017).

13 Parikh, K. *et al.* Colonic epithelial cell diversity in health and inflammatory bowel disease. *Nature* **567**, 49-55, (2019).

14 MacParland, S. A. *et al.* Single cell RNA sequencing of human liver reveals distinct intrahepatic macrophage populations. *Nat Commun* **9**, 4383, (2018).

**
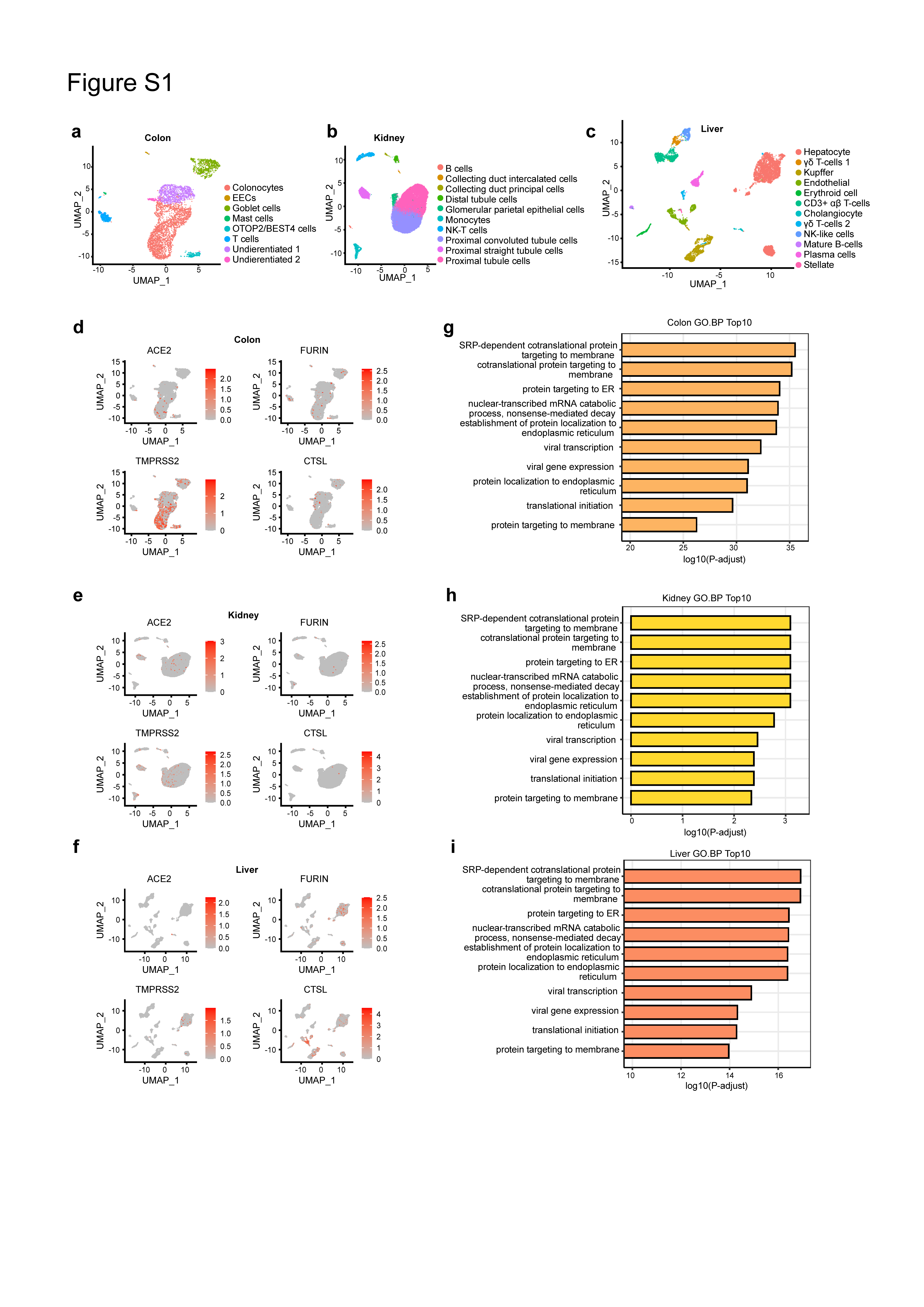
**

**Figure. S1.**

**Translation-related pathways were enriched in SARS-CoV-2 susceptible cells.** (a-c) Human colon kidney and liver single-cell transcriptional atlas. Uniform manifold approximation and projection (UMAP) plots to show different cell types identified by scRNA-seq (left). Cell identities are color-coded according to list of annotations used in all panels for the different cell types (right). (d-f) Expression of ACE2 and several candidate receptors that may facilitate SARS-CoV-2 entry. The same UMAP as in (a-c) were used to map different clusters of cell sub-populations, color density represents according gene expression level. (g-i) GO term and pathway enrichment analysis of ACE2 co-expressed genes in ACE2 positive cells from sub-populations with high ACE2 expression. For colon, the co-expression conditions are r>0.1 and p-value<0.05. For the liver, the co-expression conditions are r>0.1 and p-value<0.1. For kidney, the co-expression conditions are r>0.05 and p-value<0.1.

**
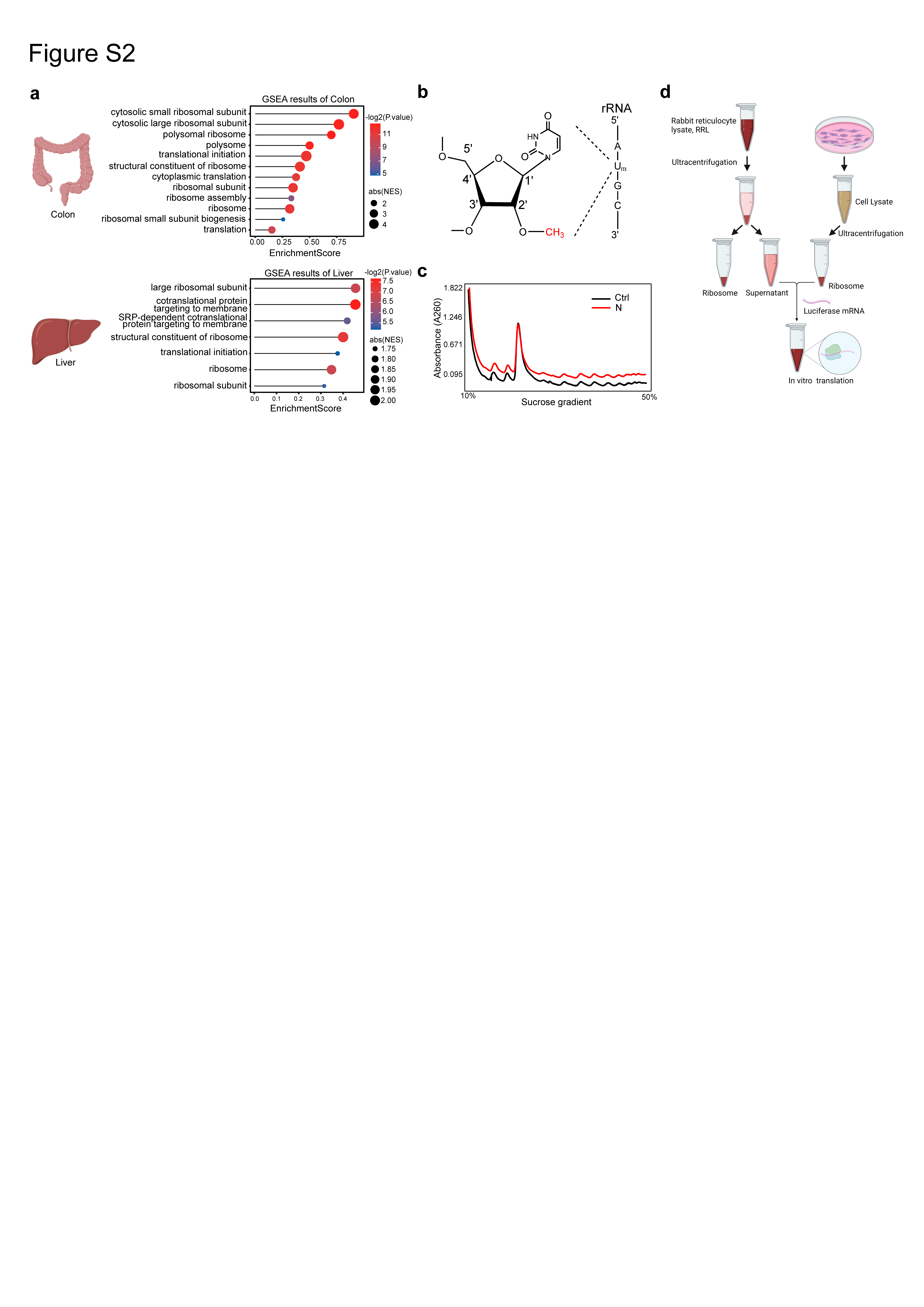
**

**Figure. S2.**

**SARS-CoV-2 N protein promoted host translation.** (a) Gene set enrichment analysis of gene expression correlation profiles of ACE2 positive cells in colonocytes from colon and cholangiocytes from liver. For colon, we selected the colonocytes with the highest ACE2 gene expression rate, screened ACE2-positive cells among them, performed correlation analysis of ACE2 in these cells with the Spearman's correlation coefficient, and screened the obtained genes (p-value<0.05) to conduct GSEA analysis. The procedure was the same for the liver, except that the cell type selected was cholangiocytes, and the p-value<0.1. (b) Schematic illustration of 2’-O-Methylated ribonucleotides, taking uracil ribonucleotide (Um) as example. The methyl group of Um was colored red. (c) Effect of SARS-CoV-2 N protein on host translation activity. 293T cells were transfected as in Fig.1d and cell lysates were centrifuged to remove debris and organelles. The supernatants were then subjected to polysome profiling to measure the overall translation activity. (d) Schematic workflow of the *in vitro* translation assay. Figures were created with BioRender.

**
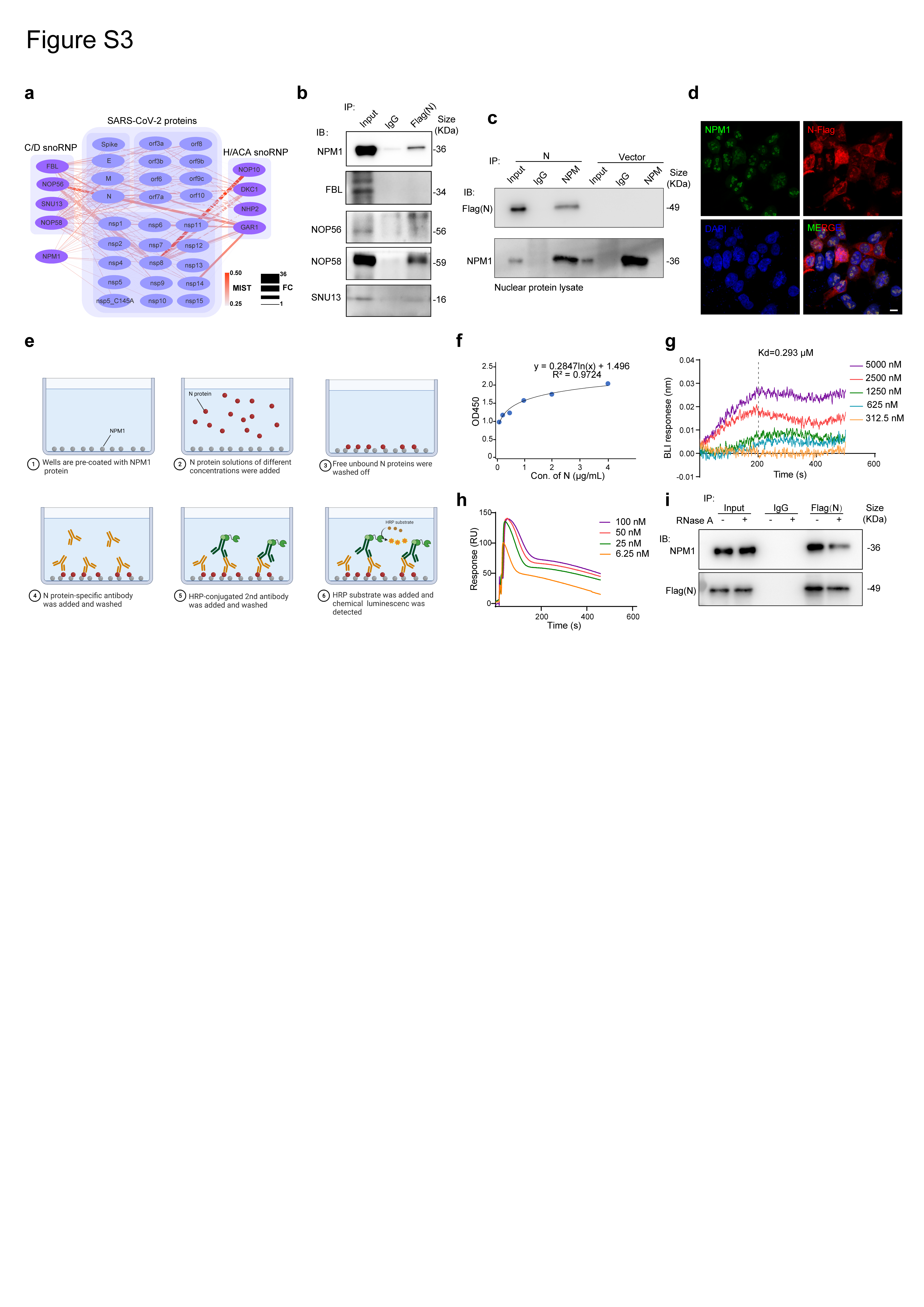
**

**Figure. S3.**

**N protein interacted with host NPM1.** (a) The interactome between SARS-CoV-2 proteins and host rRNA modification-related protein complexes is illustrated. Data from a preprint by Gordon *et al.* were re-visualized using Cytoscape software. Each colored circle represents individual protein with name in it, and lines connecting different proteins reflects the interaction between them, with width of lines reflecting fold change (FC) of enrichment over control and color darkness reflecting MIST scores. (b-d) Validation of interactions between N and host rRNA modification related proteins. 293T cells were transfected with plasmid expressing N or empty vector, and 48 h later was subjected to co-immunoprecipitation using nuclear protein lysate (b, c) or to immunofluorescence (d) experiments. Specific antibodies recognizing Flag (N, red) and NPM1 (green) were used, and DNA was stained with DAPI (blue). Scale bar, 10 μm. (e) and (f) The interaction between N protein and NPM1 was examined by ELISA. As is illustrated in (e), 96-well plate was pre-coated with NPM1, and then different concentrations of N protein were added and incubated. The interaction was examined by specific primary antibody recognizing N protein and according HRP-conjugated 2^nd^ antibody, which could generate color product by catalyzing its substrate. The correlation between concentration of N protein and OD value was fitted to logarithmic function (f). Figure (e) were created with BioRender. (g) and (h) The kinetics of interaction between N protein and NPM1 was detected by BLI assay (g) and SPR assay (h) using commercial recombinant N and NPM1 protein. (i) N protein was ectopically expressed in 293T cells. 48 hrs later, cell lysates were first treated by RNase A (100 μg/mL for 30 min), and then subjected to immunoprecipitation to examine the interaction between N and NPM1.

**
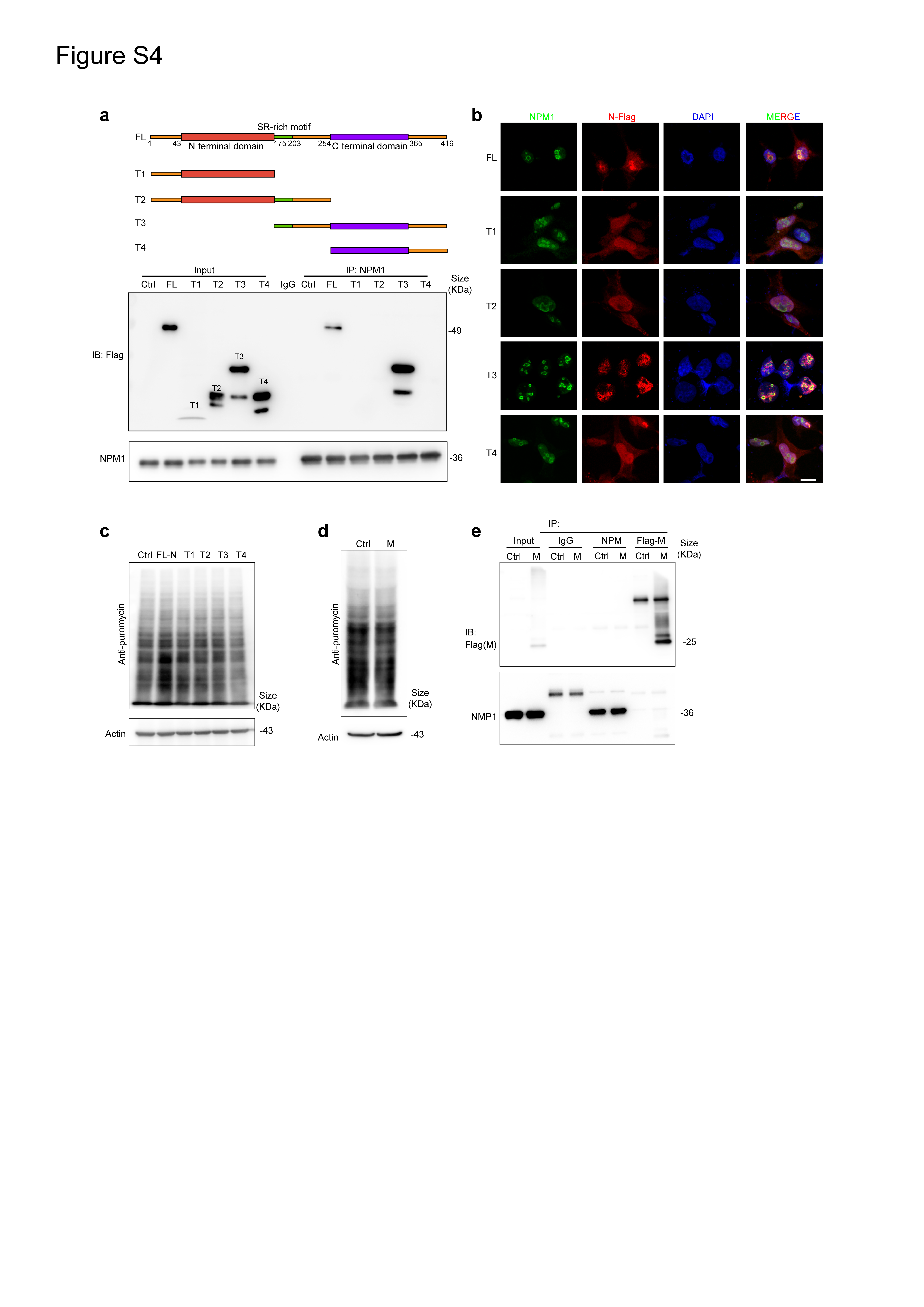
**

**Figure. S4.**

**N protein interacted with host NPM1 via SR-rich motif.** (a) and (b) Interactions between host NPM1 and different N protein truncations were assessed by co-IP and IF. Plasmid vectors expressing different N protein truncations were constructed (a, upper panel), and transfected into 293T cells. Interactions between NPM1 and different N truncations were verified by co-IP using nuclear protein lysate (a, lower panel) and IF (b). Specific antibodies recognizing Flag (N, red) and NPM1 (green) were used, and DNA was stained with DAPI (blue). Scale bar, 10 μm. N=3 independent repeats. (c) SUnSET assay showing translation activity of 293T cells that were transfected with plasmids expressing N or the different N truncations. 293T cells were transfected as in (a), and after 48 hrs were treated with puromycine (10 μg/mL) for 15 min. Cells were then lysed and overall translation activity was measured by SUnSET assay, using antibodies recognizing puromycin. (d) 293T cells transfected with plasmids expressing M protein or control. Overall translation activity was measured using SUnSET assay as in (c). (e) The interaction between SARS-CoV-2 M protein and NPM1 was tested by co-IP in 293T cells. 293T cells were transfected with plasmids expressing SARS-CoV-2 M protein, and 48 hrs later cells were lysed and total protein were extracted and subjected to immunoprecipitation using specific antibodies recognizing Flag (M) and NPM1. N=3 independent repeats.

**
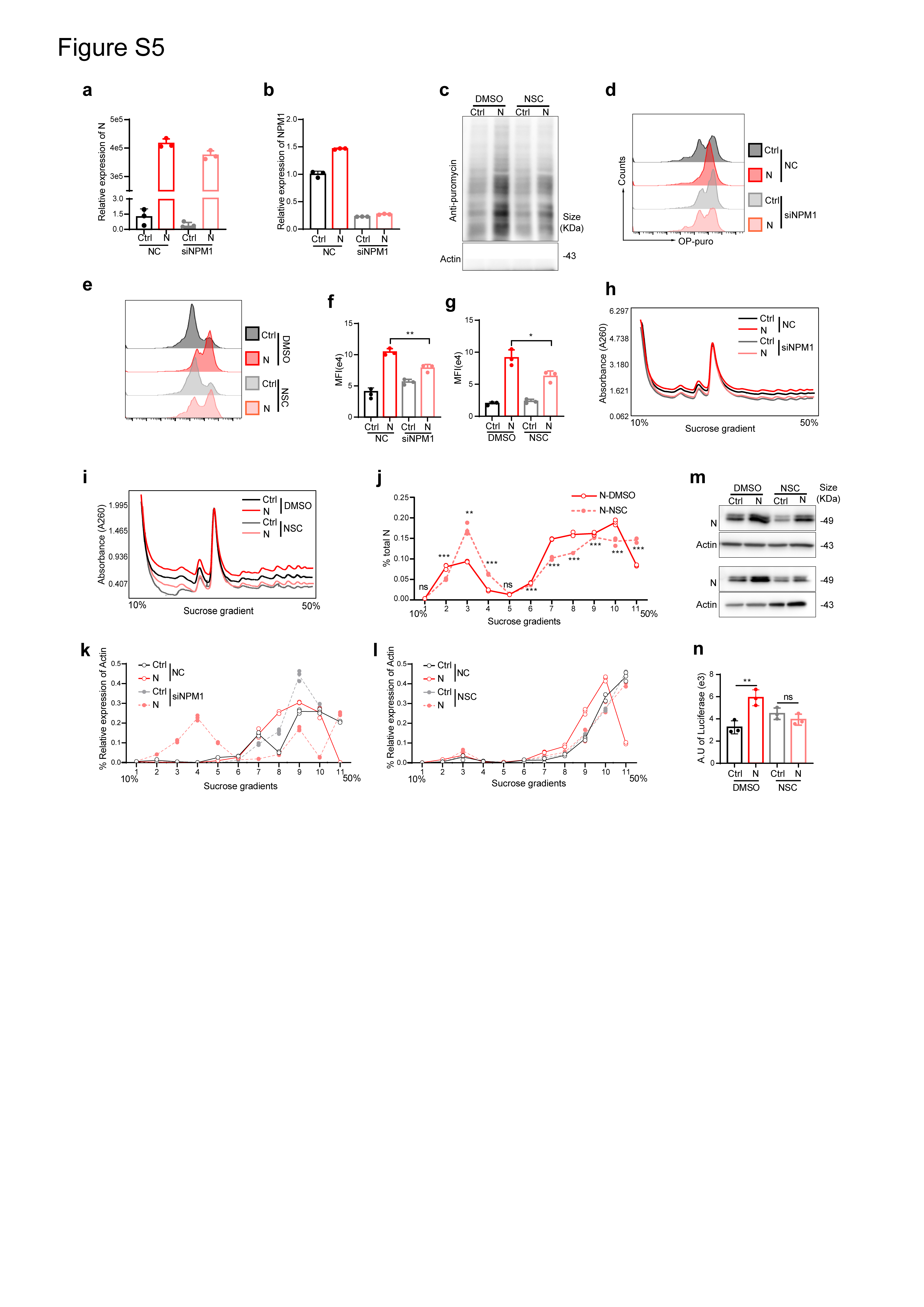
**

**Figure. S5.**

**NPM1 was indispensable for N protein function.** (a-g) Disruption of the interaction between N and NPM1 via knockdown (d) or chemical inhibition (c, e) of NPM1 attenuated translation enhancement by ectopic N expression. 293T cells were transfected with N along with NPM1 siRNA (a, b, d) or chemical inhibitor (NSC) treatment (c, e) or controls. Expressions level of N (a) and NPM1 (b) were measured by RT-qPCR after 48 hrs and overall translation activity was assessed using the SUnSET (c) and OPP-Puro (d, e) assays. (f, g) shows the statistical analysis of (d, e), respectively. (h-j) Host cell translation activity was measured by polysome profiling (h, i) and the distribution of N mRNA across these sucrose gradient fractions was examined by RT-qPCR and plotted under different treatment conditions (j). (k, l) Distribution of actin mRNA across sucrose gradient fractions was measured by RT-qPCR and plotted after NPM1 knockdown (k) or chemical inhibition of NPM1 with NSC (l). (m) and (n) ribosome translation activity was quantified using the *in vitro* translation system after chemical inhibition of NPM1. Results are presented as mean ± SD. Statistical significance of difference was calculated using un-paired student *t*-test, and error bars indicate standard deviation (**P*<0.05, ***P*<0.01, ****P*<0.001; ns, non-significant). N=3 independent repeats.


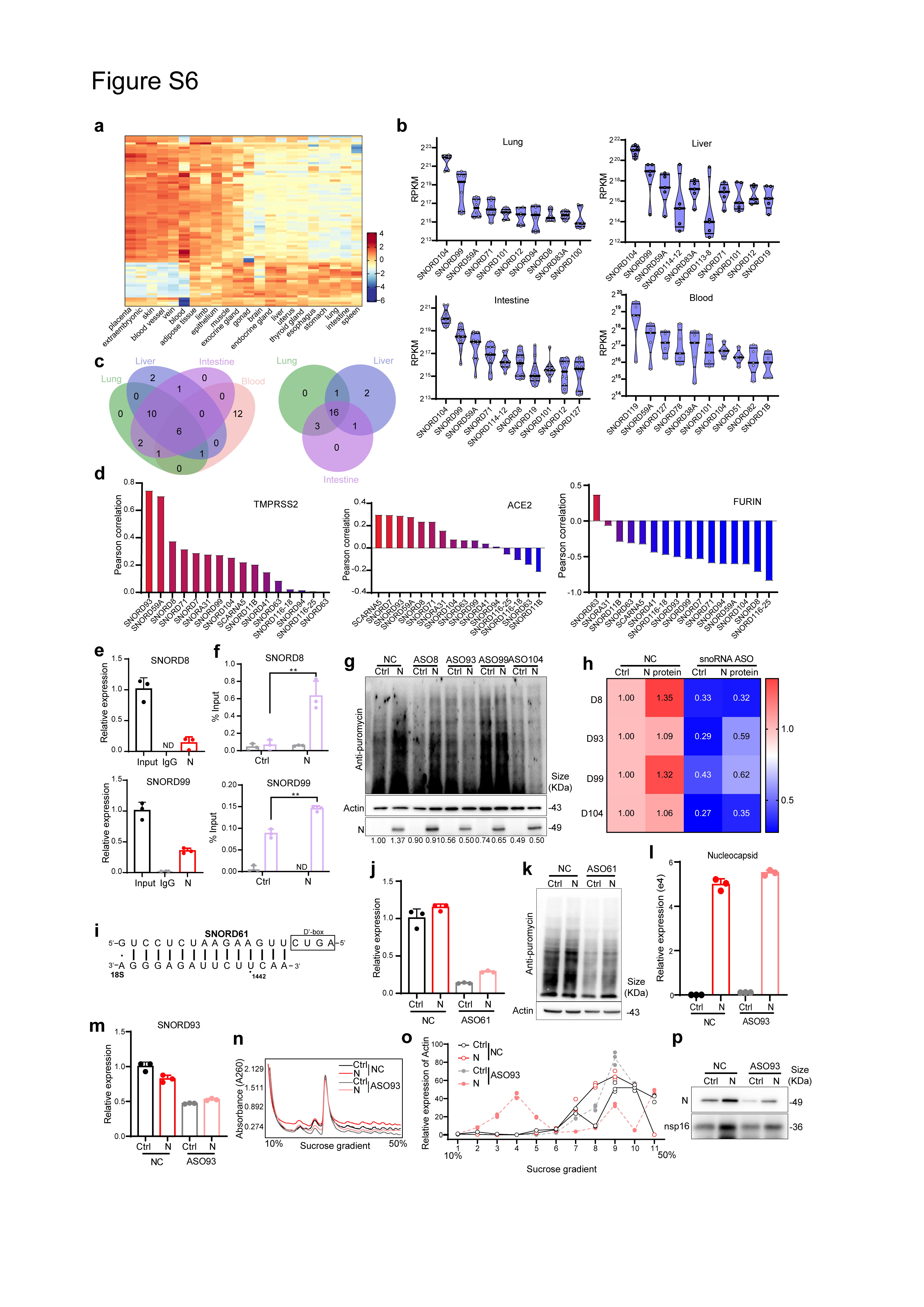


**Figure. S6.**

**N protein enhanced host translation by regulating NPM1 binding snoRNAs.** (a) Heatmap of snoRNA expression profile across different organs. Unsupervised hierarchical clustering was performed on snoRNA expression (*z*-scored log_2_ RPKM) across different tissues, utilizing small RNA-seq data from the ENCODE project. (b) Violin plot of snoRNA expression in SARS-CoV-2 susceptible (lung, liver, intestine) and unsusceptible (blood) organs. SnoRNAs were arranged according to expression levels in each organ. (c) Venn diagrams showing overlap of specific snoRNA expression among SARS-CoV-2 susceptible and unsusceptible organs/tissues. Top 20 snoRNAs with highest expression in specific organs were listed, utilizing small RNA-seq data from the ENCODE project. Left panel shows snoRNA expression overlap of these lists among SARS-CoV-2 susceptible organs (lung, intestine, liver) and unsusceptible tissue (blood), while the right panel shows overlaps among susceptible organs. (d) Correlations of expression levels between snoRNAs and SARS-CoV-2 infection-related genes. Pearson correlation *r* was calculated between snoRNA and SARS-CoV-2 infection-related genes across all organs, and snoRNAs were arranged according to *r*-value. (e) and (f) 293T cells were transfected with plasmids expression N protein. 48 hrs later, cells were lysed and RNA immunoprecipitation was performed to detect snoRNAs bond to N (e) or NPM1 (f), using antibodies targeting N, NPM1 or IgG. RNA binding was quantified by RT-qPCR and shown as relative enrichment to input (10% loading). (g) 293T cells were transfected with N-expressing plasmids and snoRNA antisense oligos (ASOs), and cells were lysed and translation activity was measured using SUnSET after 48 hrs. (h) Heatmap showing expression of snoRNAs under different treatment conditions. 293T cells were transfected with plasmids expressing N or empty vector, along with specific snoRNA ASOs. snoRNA expression was measured by RT-qPCR after 48 hrs. (i) Sequence alignment between snoRNA U61 and its target predicted using snoRNA Atlas. (j) and (k) 293T cells were transfected with plasmids expressing N, along with snoRNA U61 ASO. U61 level was examined by RT-qPCR (j), and host translation activity after transfection was detected by SUnSET assay (k). (l) and (m) 293T cells were transfected with plasmid expressing N and SNORD93 ASO, and expression of N (l) and SNORD93 (m) were examined by RT-qPCR. (n) and (o) 293T cells were transfected with N-expressing plasmids and SNORD93 ASO, and polysome profiling was performed to detect global translation activity (n). Distribution of actin mRNA across sucrose gradient fractions measured by RT-qPCR and plotted after knockdown of SNORD93 (o). (p) Translation efficiency of nsp16 mRNA was examined using the *in vitro* translation system. N protein were ectopically expressed and SNORD93 was knocked down in 293T cells, and ribosomes were isolated. *In vitro* translation assay was performed to quantify translation activity. *In vitro* transcribed N and nsp16 mRNAs were added to evaluate ribosome translation efficiency. N=3 independent repeats.

**
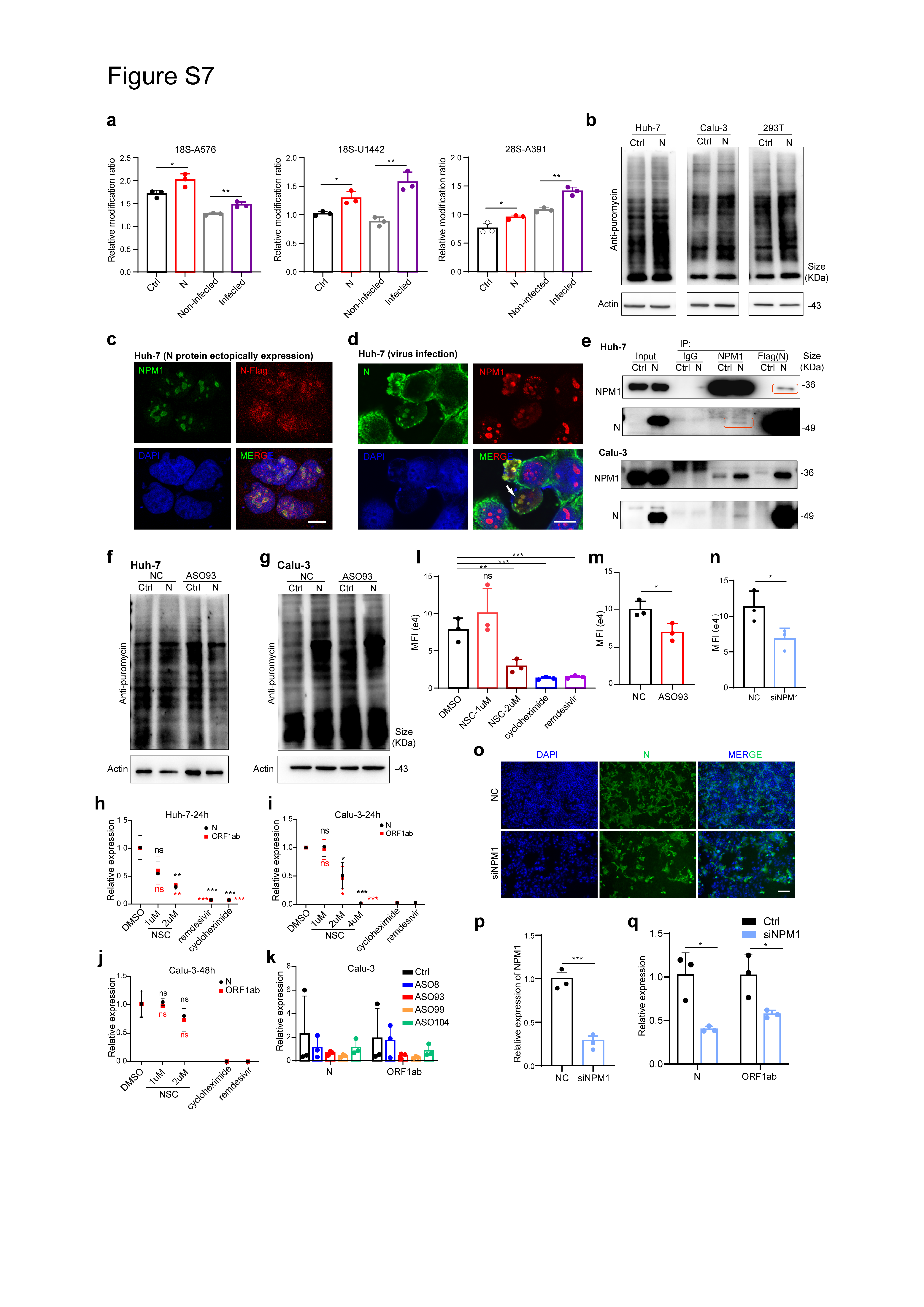
**

**Figure. S7.**

**NPM1 inhibition and snoRNA-targeted therapies ameliorated SARS-CoV-2 proliferation *in vitro*.** (a) 2’-O-Me modification levels at specific sites (18S-A576, 18S-U1442, 28S-A391)were detected by RTL-P assay after overexpression of N protein in 293T cells or SARS-CoV-2 infection in Vero 6 cells. (b) Huh-7 and Calu-3 cells were transfected with plasmids expressing N and global translation activity was assessed with the SUnSET assay. 293T cells were treated in parallel for comparison. (c-e) Huh-7 and Calu-3 cells were treated as in (b), and the interaction between N and NPM1 was verified by IF co-localization (c) and co-IP (e). Huh-7 cells were infected with SARS-CoV-2 at an MOI of 0.01, and the interaction was verified by IF (d). Scale bar, 10 μm. (f) and (g) Huh-7 and Calu-3 cells were transfected with plasmids expressing N and SNORD93 ASO simultaneously and translation activity was detected by SUnSET assays. (h-j) Huh-7 and Calu-3 cells were infected with SARS-CoV-2 at an MOI of 0.01 and treated with NPM1 inhibitor (NSC), remdesivir, or cycloheximide, and SARS-CoV-2 genomic RNA in the culture medium was quantified by RT-qPCR at 24 and 48 hpi. (k) Calu-3 cells were infected with SARS-CoV-2 at an MOI of 0.01 and transfected with snoRNA ASOs, and SARS-CoV-2 genomic RNA in the culture medium was quantified by RT-qPCR at 48 hpi. (l) and (m) Statistical results of fluorescence intensity in Fig.1v and 1w. (n-q) Huh-7 cells were infected with SARS-CoV-2 at an MOI of 0.01 and NPM1 was knocked down by siRNA. Virus proliferation was detected by immunostaining (n) and (o) and RT-qPCR (p). Expression of NPM1 after transfection of siRNA was verified by RT-qPCR (p). Statistical significance of difference was calculated using un-paired student *t*-test, and error bars indicate standard deviation (**P*<0.05, ***P*<0.01, ****P*<0.001; ns, non-significant).

**
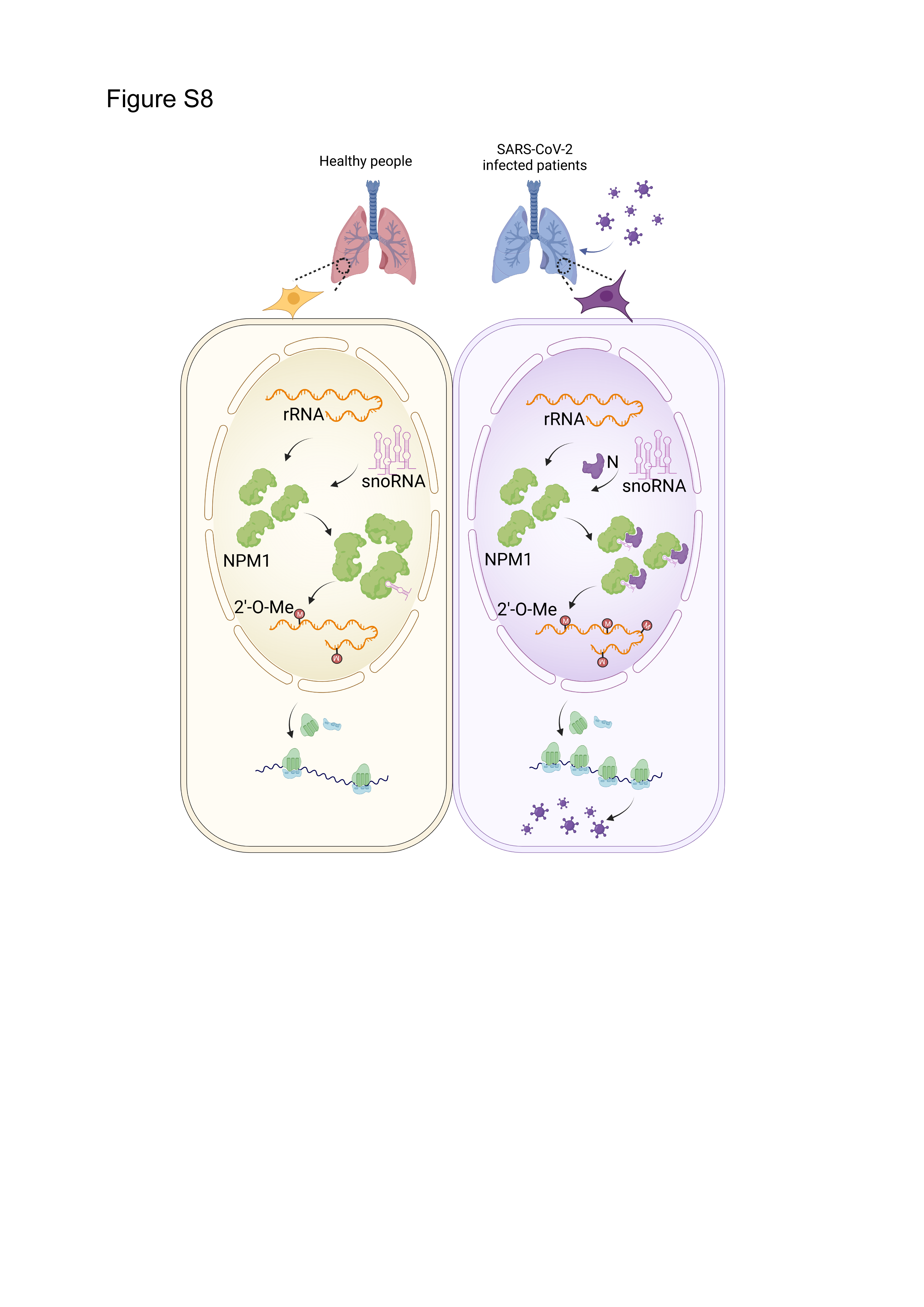
**

**Figure. S8.**

**Graphical illustration of effects of SARS-CoV-2 N protein on host translation machinery and according mechanisms.** Created with BioRender.com.

**Table S1.**

**Source and identifiers**

| REAGENT or RESOURCE | SOURCE | IDENTIFIER |
| --- | --- | --- |
| Antibodies |  |  |
| anti-SARS-CoV-2 Nucleocapsid | Sino Biological | Cat#40143 |
| anti-Puromycin Antibody, clone 12D10 | Millipore | Cat#MABE343 |
| anti-NPM1 | Sigma | Cat#B0556 |
| anti-FBL | Cell Signaling Technology | Cat#2639S |
| anti-SNU13 | Abclonal | Cat#A5926 |
| anti-NOP56 | Abclonal | Cat#A8040 |
| anti-NOP58 | Abclonal | Cat#A4749 |
| anti-Flag (goat) | Abcam | Cat#ab1257 |
| anti-Flag (mouse) | Sangon Biotech | Cat#D191041 |
| Alexa Fluor488®-conjugated Goat Anti-rabbit IgG | Invitrogen | Cat#A11034 |
| anti-Flag(rabbit) | Sangon Biotech | Cat#D110005 |
| Chemicals and commercial kits | | |
| Glycoblue | Thermo Fisher Scientific | Cat#AM9516 |
| Antarctic Phosphatase | NEB | Cat#M0289S |
| T4 PNK | NEB | Cat#M0201L |
| ATP | Sangon Biotech | Cat#R0441 |
| Rneasy MinElute Cleanup kit | QIAGEN | Cat#74104 |
| Rabbit reticulocyte lysate, Nuclease-Treated | Promega | Cat#L4960 |
| Puromycin | Sangon Biotech | Cat#A610593 |
| RNA Immunoprecipitation kit | BersinBio | Cat#Bes5101 |
| Click-iT Plus OPP Protein Synthesis Assay Kit | Thermo Fisher Scientific | Cat#C10456 |
| 2019-nCoV qRT-PCR kit | Liferiver | Cat# RR-0479-02 |
| Cycloheximide | Sigma | Cat#C7698 |
| RNase inhibitor | Thermo Fisher Scientific | Cat#AM2694 |
| SARS-CoV-2 (2019-nCoV) Nucleocapsid-His recombinant Protein | Sino Biological | Cat#40588-V08B |
| Recombinant human NPM1 protein | Sino Biological | Cat#10053-H07E1 |
| HRP substrate | InnoReagents | Cat# EL0005 |
| blocking reagent | InnoReagents | Cat#EL0153 |
| Cytoplasmic and nuclear protein extraction kit | Beyotime | Cat#P0028 |
| coating buffer | InnoReagents | Cat#EL0155 |
| Protease inhibitor cocktail | Thermo Fisher Scientific | Cat#78430 |
| HiScript II reverse transcriptase | Vazyme | Cat#R223-00 |
| HiScribe T7 High Yield RNA Synthesis Kit | NEB | Cat#E2050S |
| Universal SYBR Green Supermix | Bio-Rad | Cat#1725270 |
| Experimental Models: Cell Lines | |  |
| HEK293T | ATCC | Cat#CRL-3216 |
| Calu-3 | ATCC | Cat#HTB-55 |
| Huh-7 | Japanese Collection of Research Bioresources | Cat# JCRB0403 |
| Oligonucleotides |  |  |
| RT-qPCR SARS-CoV-2 N Forward: GGGGAACTTCTCCTGCTAGAAT | SunYa | N/A |
| RT-qPCR SARS-CoV-2 N Reverse: CAGACATTTTGCTCTCAAGCTG | SunYa | N/A |
| RT-qPCR NPM1 Forward: GCCAGTGCATATTAGTGGACAGC | SunYa | N/A |
| RT-qPCR NPM1 Reverse: GGAACCTTGCTACCACCTCCAG | SunYa | N/A |
| RT-qPCR U6 Forward: CTCGCTTCGGCAGCACA | SunYa | N/A |
| RT-qPCR U6 Reverse: AACGCTTCACGAATTTGCGT | SunYa | N/A |
| RT-qPCR actin Forward: CCTCGCCTTTGCCGATCC | SunYa | N/A |
| RT-qPCR actin Reverse: GGATCCTTCATGAGGTAGTCAGTC | SunYa | N/A |
| RT-qPCR SNORD8 Forward: CCCAATGATGAGTTGCCATG | SunYa | N/A |
| RT-qPCR SNORD8 Reverse: CCCTCAGATCTTCATGTGAGAAG | SunYa | N/A |
| RT-qPCR SNORD93 Forward: TGGCCAAGGATGAGAACTCT | SunYa | N/A |
| RT-qPCR SNORD93 Reverse: TGGCCTCAGGTAAATCCTTTA | SunYa | N/A |
| RT-qPCR SNORD99 Forward: TGGTCCAGGATGAAACCTAA | SunYa | N/A |
| RT-qPCR SNORD99 Reverse: CCTAGGAGCTGGTCTCAGTCC | SunYa | N/A |
| RT-qPCR SNORD104 Forward: CCTGCTGTGATGACATTC | SunYa | N/A |
| RT-qPCR SNORD104 Reverse: CAGGCTCAGACTCCAGTT | SunYa | N/A |
| RT-qPCR SNORD61 Forward: GCTATGATGAATTTGATTGCATTG | SunYa | N/A |
| RT-qPCR SNORD61 Reverse: AAGCTCAGAACTTCTTAGAGGACA | SunYa | N/A |
| RTL-P 18S-576 Forward1: TCGAGGCCCTGTAATTGGA | SunYa | N/A |
| RTL-P 18S-576 Forward2: TCCATTGGAGGGCAAGTCT | SunYa | N/A |
| RTL-P 18S-576 Reverse: AACTGCAGCAACTTTAATATACGCT | SunYa | N/A |
| RTL-P 18S-1442 Forward1: GACTCTGGCATGCTAACTAGTTA | SunYa | N/A |
| RTL-P 18S-1442 Forward2: ACAAGTGGCGTTCAGCCA | SunYa | N/A |
| RTL-P 18S-1442 Reverse: GCAGCCCCGGACATCTAA | SunYa | N/A |
| RTL-P 28S-391 Forward1: ATAGTCAACAAGTACCGTAAGGGAA | SunYa | N/A |
| RTL-P 28S-391 Forward2: GGCGTGAAACCGTTAAGAG | SunYa | N/A |
| RTL-P 28S-391 Reverse: TTGAATCCTCCGGGCGGA | SunYa | N/A |
| SNORD8 ASO: CAGUAttagcatggcAACUC | GenePharma | N/A |
| SNORD93 ASO: AGCACataaaatcagAUUAG | GenePharma | N/A |
| SNORD99 ASO: CAGUCccatatccgcAUUUC | GenePharma | N/A |
| SNORD104 ASO: GCAGUctaacacgtgCUUUA | GenePharma | N/A |
| SNORD61 ASO: CUCAGaacttcttagAGGAC | GenePharma | N/A |
| Recombinant DNA |  |  |
| pcDNA6B-nCoV-N-FLAG | Dr. Peihui Wang | N/A |
| pcDNA6B-nCoV-M-FLAG | Dr. Peihui Wang | N/A |
| pcDNA6B-nCoV-N truncation 1-FLAG | This study | N/A |
| pcDNA6B-nCoV-N truncation 2-FLAG | This study | N/A |
| pcDNA6B-nCoV-N truncation 3-FLAG | This study | N/A |
| pcDNA6B-nCoV-N truncation 4-FLAG | This study | N/A |

**Table S2.**

**Encode IDs of data used for snoRNA expression**

| tissue | ENCODE ID |
| --- | --- |
| placenta | ENCFF039AID |
| placenta | ENCFF051YNI |
| placenta | ENCFF381JLQ |
| placenta | ENCFF400SCX |
| placenta | ENCFF834GRE |
| placenta | ENCFF998YBK |
| extraembryonic-component | ENCFF039AID |
| extraembryonic-component | ENCFF051YNI |
| extraembryonic-component | ENCFF232RYL |
| extraembryonic-component | ENCFF381JLQ |
| extraembryonic-component | ENCFF400SCX |
| extraembryonic-component | ENCFF653RKY |
| extraembryonic-component | ENCFF673VYV |
| extraembryonic-component | ENCFF834GRE |
| extraembryonic-component | ENCFF970ZBS |
| extraembryonic-component | ENCFF998YBK |
| skin | ENCFF060SLT |
| skin | ENCFF084WOZ |
| skin | ENCFF111BZI |
| skin | ENCFF296CFQ |
| skin | ENCFF364TXK |
| skin | ENCFF411KQN |
| skin | ENCFF491QWS |
| skin | ENCFF508ILI |
| skin | ENCFF550QHO |
| skin | ENCFF605QXI |
| skin | ENCFF631YQD |
| skin | ENCFF659FNV |
| skin | ENCFF705UBV |
| skin | ENCFF726NYB |
| skin | ENCFF846NDU |
| skin | ENCFF967LTP |
| blood-vessel | ENCFF027NAH |
| blood-vessel | ENCFF221LZG |
| blood-vessel | ENCFF304UWW |
| blood-vessel | ENCFF494VCO |
| blood-vessel | ENCFF781PVR |
| blood-vessel | ENCFF845VHL |
| blood-vessel | ENCFF922RFW |
| blood-vessel | ENCFF974IUZ |
| vein | ENCFF304UWW |
| vein | ENCFF781PVR |
| vein | ENCFF845VHL |
| vein | ENCFF922RFW |
| blood | ENCFF240JNA |
| blood | ENCFF243BUN |
| blood | ENCFF399NBS |
| blood | ENCFF640YKF |
| blood | ENCFF658GLX |
| adipose-tissue | ENCFF607DEC |
| adipose-tissue | ENCFF735BGE |
| adipose-tissue | ENCFF886QPG |
| adipose-tissue | ENCFF940IVG |
| limb | ENCFF033GUS |
| limb | ENCFF307CKR |
| limb | ENCFF316XWT |
| epithelium | ENCFF008YAE |
| epithelium | ENCFF046IHJ |
| epithelium | ENCFF051YNI |
| epithelium | ENCFF143PXA |
| epithelium | ENCFF221LZG |
| epithelium | ENCFF283EBP |
| epithelium | ENCFF304UWW |
| epithelium | ENCFF364TXK |
| epithelium | ENCFF400SCX |
| epithelium | ENCFF491QWS |
| epithelium | ENCFF550QHO |
| epithelium | ENCFF561FEI |
| epithelium | ENCFF726NYB |
| epithelium | ENCFF781PVR |
| epithelium | ENCFF787UEM |
| epithelium | ENCFF845VHL |
| epithelium | ENCFF846NDU |
| epithelium | ENCFF922RFW |
| epithelium | ENCFF967LTP |
| epithelium | ENCFF974IUZ |
| epithelium | ENCFF985VTV |
| muscle | ENCFF023FHD |
| muscle | ENCFF071SUO |
| muscle | ENCFF260CUI |
| muscle | ENCFF271ZWH |
| muscle | ENCFF327KWU |
| muscle | ENCFF335UXC |
| muscle | ENCFF395JEC |
| muscle | ENCFF479DML |
| muscle | ENCFF491FVE |
| muscle | ENCFF511OQS |
| muscle | ENCFF781PLK |
| muscle | ENCFF805EHA |
| muscle | ENCFF813EOM |
| exocrine-gland | ENCFF008YAE |
| exocrine-gland | ENCFF046IHJ |
| exocrine-gland | ENCFF211IKV |
| exocrine-gland | ENCFF283EBP |
| exocrine-gland | ENCFF836FKY |
| exocrine-gland | ENCFF870SDO |
| gonad | ENCFF231MYQ |
| gonad | ENCFF361BBQ |
| gonad | ENCFF419DBG |
| brain | ENCFF080HGZ |
| brain | ENCFF122QGO |
| brain | ENCFF212YYE |
| brain | ENCFF359SIY |
| brain | ENCFF500FRR |
| brain | ENCFF515YKZ |
| brain | ENCFF520VCD |
| brain | ENCFF617OIT |
| brain | ENCFF733ZEX |
| brain | ENCFF743GIP |
| brain | ENCFF763TMM |
| brain | ENCFF912MJP |
| endocrine-gland | ENCFF046IHJ |
| endocrine-gland | ENCFF108AWH |
| endocrine-gland | ENCFF148AXN |
| endocrine-gland | ENCFF211IKV |
| endocrine-gland | ENCFF267WSV |
| endocrine-gland | ENCFF283EBP |
| endocrine-gland | ENCFF777QLW |
| endocrine-gland | ENCFF836FKY |
| endocrine-gland | ENCFF853RZQ |
| endocrine-gland | ENCFF870SDO |
| Liver | ENCFF046IHJ |
| Liver | ENCFF211IKV |
| Liver | ENCFF283EBP |
| Liver | ENCFF836FKY |
| Liver | ENCFF870SDO |
| uterus | ENCFF664GDW |
| uterus | ENCFF811ONI |
| uterus | ENCFF979TYQ |
| thyroid-gland | ENCFF108AWH |
| thyroid-gland | ENCFF148AXN |
| thyroid-gland | ENCFF267WSV |
| esophagus | ENCFF071SUO |
| esophagus | ENCFF143PXA |
| esophagus | ENCFF479DML |
| esophagus | ENCFF561FEI |
| esophagus | ENCFF781PLK |
| esophagus | ENCFF787UEM |
| esophagus | ENCFF985VTV |
| stomach | ENCFF271ZWH |
| stomach | ENCFF327KWU |
| stomach | ENCFF483QVT |
| stomach | ENCFF491FVE |
| stomach | ENCFF546RWF |
| stomach | ENCFF647XYE |
| stomach | ENCFF813EOM |
| stomach | ENCFF827WAI |
| Lung | ENCFF114VZQ |
| Lung | ENCFF208DUJ |
| Lung | ENCFF219PSK |
| Lung | ENCFF275QHE |
| Lung | ENCFF590YHJ |
| Lung | ENCFF849GKQ |
| intestine | ENCFF233DTP |
| intestine | ENCFF260HKU |
| intestine | ENCFF269LNT |
| intestine | ENCFF410IZL |
| intestine | ENCFF429VLR |
| intestine | ENCFF668PIG |
| intestine | ENCFF700MJH |
| intestine | ENCFF710JYW |
| intestine | ENCFF803RFD |
| intestine | ENCFF980YWZ |
| spleen | ENCFF183GYF |
| spleen | ENCFF223RVK |
| spleen | ENCFF948LKQ |
| spleen | ENCFF972HOG |
